# Genetic predisposition to smoking in relation to the risk of frailty in ageing

**DOI:** 10.1038/s41598-023-28780-0

**Published:** 2023-02-10

**Authors:** Wei Liu, Hong Yang, Linshuoshuo Lv, Jie Song, Yuqing Jiang, Xiaohui Sun, Ding Ye, Yingying Mao

**Affiliations:** Department of Epidemiology, School of Public Health, Zhejiang Chinese Medical University, Hangzhou, 310053 China

**Keywords:** Genetic association study, Risk factors, Epidemiology, Genetics research

## Abstract

Frailty causes emerging global health burden due to its high prevalence and mortality. In this study, we used Mendelian randomization (MR) approach to examine the potential causal relationship between smoking and frailty in ageing. Using inverse-variance weighted (IVW) method, genetically predicted smoking initiation was associated with an increased risk of frailty in ageing (odd ratio (OR) 1.23, 95% confidence interval (CI) 1.19–1.27, *P* = 3.21 × 10^–39^). Similarly, per year increase in age of initiation of regular smoking was associated with a 25% decrease in the risk of frailty (95% CI 7–39%, *P* = 7.79 × 10^–3^, per year), while higher number of cigarettes per day was associated with a 12% increased risk (95% CI 4–20%, *P* = 1.76 × 10^–3^). Compared with former smokers, current smokers were associated with an increased risk of frailty (OR 1.12, 95% CI 1.02–1.22, *P* = 0.01). Lifetime smoking was associated with a 46% higher risk of frailty (95% CI 37–56%, *P* = 2.63 × 10^–29^). Sensitivity analysis using alternative MR methods yielded similar results. Our study indicates that genetic predisposition to smoking is associated with the risk of frailty in ageing. Further studies are warranted to examine the exact role of smoking in the development of frailty.

## Introduction

Frailty is a clinical status which individuals are more likely to have negative health-related events when expose to exogenous or endogenous pathogenic factors^1^. It is characterized by the decline of physiological function along with the increased vulnerability to stressors. Frailty occurs with ageing and become a high-risk factor for various adverse health outcomes, such as falls, hospitalization, and death^2^. A common way to measure frailty is to calculate the frailty index based on the proportion of measured health deficits accumulated by individuals with age^3^. A meta-analysis has reported that in western countries, the prevalence rate of frailty was 26.8% (95% confidence interval (CI) 22.1–31.5%) in 56,407 older people averaged 78.59 years old^4^. Compared with normal older adults, the average relative increase in mortality risk was 15% in older adults aged over 65 years old with frailty^5^. Since frailty is a reversible clinical syndrome, identifying its risk factors and taking precautions against it can effectively prevent the occurrence of the syndrome and reduce the globe medical burden.

Smoking is associated with several negative health-related events, such as respiratory diseases and cardiovascular disease. Observational epidemiological studies generally support a positive association between smoking and the risk of frailty in ageing. For example, a cohort study including 261 current smokers and 2281 non-smokers of community-dwelling older people aged over 60 years old showed that current smokers were twice as likely to develop frailty compared with non-smokers (odd ratio (OR) 2.07, 95% CI 1.39–3.39, *P* = 0.001)^6^. However, another cohort study including 2573 short-term follow-up participants and 1309 long-term follow-up participants identified that smoking was not associated with the risk of frailty (OR 0.93, 95% CI 0.48–1.79 for short-term follow-up; OR 0.92, 95% CI 0.45–1.86 for long-term follow-up)^7^. These conflicting results from conventional observational studies may be due to differences in the study population, study design, as well as bias inherent in observational studies such as confounding and reverse causality, which weakens the reliability of the results to a certain extent.

Mendelian randomization (MR) is an genetic epidemiologic method which uses genetic variants as instrumental variables (IVs) to determine whether an observational association between exposures and outcomes is consistent with a causal effect^8^. MR is based on Mendel’s second law of independences which suggests that the inheritance of one trait is independent of the inheritance of other traits^9^. MR can reduce bias by confounding factors because alleles are presumed to be randomly allocated at conception and meiosis yields a random distribution of genetic variants in a population. Meanwhile, it can avoid reverse causation because diseases cannot affect genotype^10^. Therefore, in the present study, we aimed to examine the association between smoking and frailty by using a two-sample MR design.

## Material and methods

### Outcome data sources

**Figure 1:**
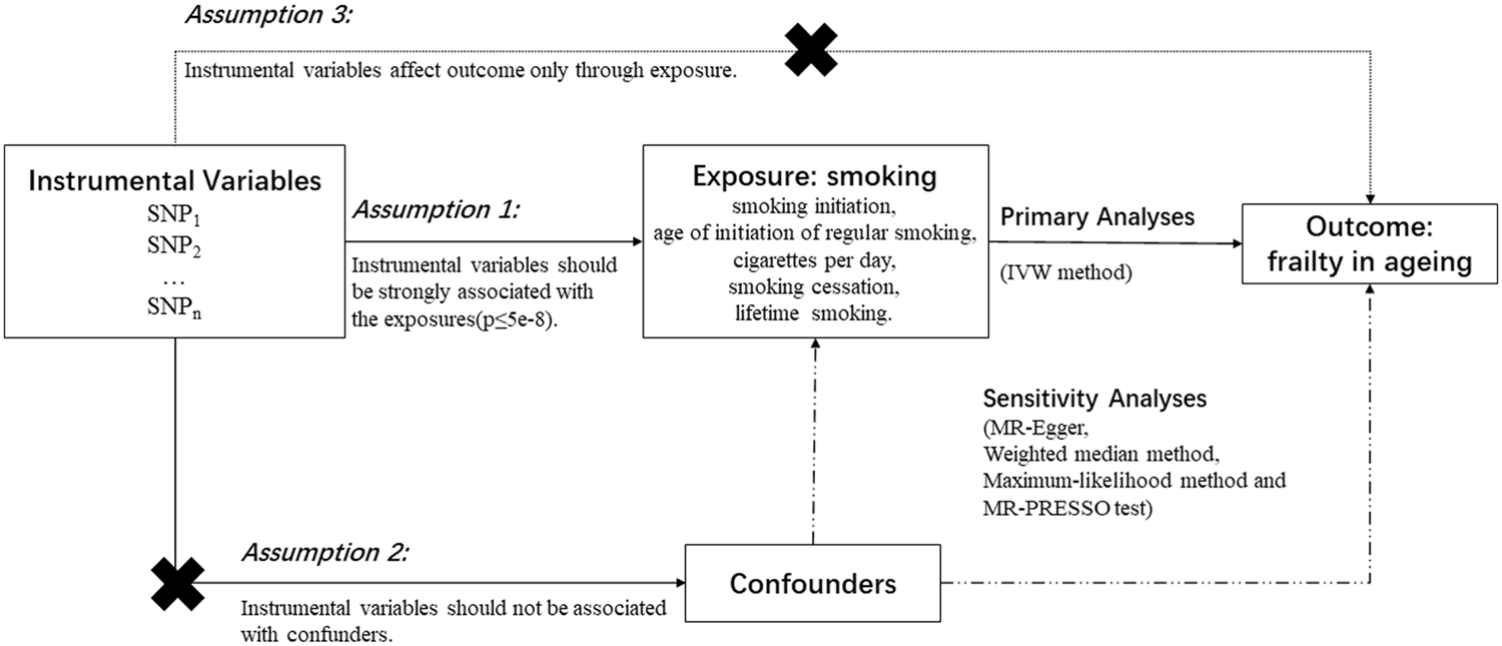
An overview of the study design.

An overview of the study design is shown in Fig. 1. Genetic association data of frailty in ageing were obtained from a meta-analysis of genome-wide association studies (GWASs) from the UK Biobank (n = 164,610 participants) and Swedish TwinGene (n = 10,616 participants). The participants of the UK Biobank were European populations aged 60 to 70 years with 51.3% females, and the participants of TwinGene were European descents aged 41 to 87 years and 52.5% of them are females. Frailty index was calculated by 49 or 44 self-reported items on healthy deficits for the UK Biobank and TwinGene, respectively. The genotyping of the UK Biobank and TwinGene was performed using custom Affymetrix microarrays and Illumina OmniExpress platform, respectively. 1000 Genomes project and the Haplotype Reference Consortium (HRC) reference panels were used for imputation. A total of 14 loci were identified to be associated with frailty index (*P* < 5 × 10^–8^), and the single nucleotide polymorphism (SNP)-based heritability was estimated to be 11% by Linkage Disequilibrium Score Regression (LDSR)^11^.

### Selection of instrumental variables

IVs for smoking related phenotypes including smoking initiation (SmkInit), age of initiation of regular smoking (AgeSmk), cigarettes per day (CigDay), smoking cessation (SmkCes), and lifetime smoking, were obtained from a recently published GWAS and a GWAS meta-analyses.

Specifically, for SmkInit, AgeSmk, CigDay and SmkCes, IVs were obtained from a meta-analysis on tobacco and alcohol consumption, which identified 364, 10, 47 and 22 independent genetic variants associated with these four smoking phenotypes (*P* < 5 × 10^–8^) in over 1,232,091 individuals of European descent, respectively^12^. SmkInit (n = 1,232,091) and AgeSmk (n = 341,427) indicated whether an individual had ever smoked regularly. Heaviness of smoking was measured with CigDay (n = 337,334), and SmkCes (n = 547,219) was a binary variable contrasting current versus former smokers. IVs for lifetime smoking were selected from a GWAS from the UK Biobank which involved 462,690 European descent^13^. Lifetime smoking was combined by the smoking measures and a simulated half-life constant. A total of 126 independent genetic variants associated with lifetime smoking were used as IVs (*P* < 5 × 10^–8^).

The definitions of each smoking related phenotype have been described in detail elsewhere^12,13^, and relevant information about smoking related phenotypes are listed in Supplementary Table 1.

However, two genetic variants of SmkInit (rs2587507 and rs3076896) and one genetic variant of SmkCes (rs12203592) were not available in the summary data for frailty. Therefore, a total of 362, 10, 47, 21 and 126 genetic variants associated with SmkInit, AgeSmk, CigDay, SmkCes and lifetime smoking were used as IVs in this study, respectively. All the genetic variants were clumped to ensure independence at linkage disequilibrium (LD) r^2^ < 0.1. Detailed information of the IVs used are presented in Supplementary Table 2.

Since the data used in the present study were based on published studies and public databases, no additional ethical approval from the institutional review board was required.

### Statistical analysis

All statistical analyses were performed using “MendelianRandomization”^14^ and “MRPRESSO”^15^ packages in R software version 4.1.2, unless otherwise noted. F-statistics were calculated to assess the strength of IVs. F-statistics were constructed based on the formula as follow^16^: F-statistics = ${\text{R}}^{2} \times ({\text{N}} - 1 - {\text{k}})/((1 - {\text{R}}^{2} ) \times {\text{k}})$, where R^2^ is the phenotypic variability explained by the instrument, N is the number of people exposure, and k is the number of SNPs used as IVs. An F-statistics > 10 indicates that the IVs used are less likely to suffer from weak instrument bias.

The primary MR analysis was conducted using a inverse-variance weighted (IVW) method, with the supplement of weighted median method^17^ and maximum-likelihood method^18^. IVW method combines the Wald ratio estimates of each genetic variants into one causal estimate for each risk factor^19^. Cochran’s Q statistics and I^2^ was performed to assess the heterogeneity across individual SNPs. *P*-heterogeneity < 0.05 and I^2^ > 50% indicates the presence of heterogeneity. When *P*-heterogeneity > 0.05, I^2^ < 50%, we used fixed effect model, otherwise, the random effect model was used. For the weighted median method is even when up to 50% of information comes from invalid IVs, the estimator will still be consistent. Maximum-likelihood method is used to estimate the causal effect by direct maximization of the likelihood given the genetic variants effects of exposure and outcome which assumes a linear relationship between the exposure and the outcome.

MR-Pleiotropy RESidual Sum and Outlier (MR-PRESSO) test^14^ and MR-Egger regression^20^ were used to test for potential outliers and pleiotropy. MR-PRESSO test evaluates horizontal pleiotropy in multi-instrument summary level. MR-PRESSO global test evaluates overall horizontal pleiotropy amongst all IVs in a single MR test by comparing the observed distance of all the variants to the regression line (residual sum of squares) to the expected distance under the null hypothesis of no horizontal pleiotropy. MR-PRESSO test evaluates the presence of specific horizontal pleiotropic outlier variants by using the observed and expected distributions of the tested variant. MR-Egger regression is under an assumption that the association of each genetic variant with the exposure is independent of the pleiotropic effect of the variant, which gives a valid test of the null causal hypothesis and a consistent causal effect estimate even when all the genetic variants are invalid IVs. The *P*-value for the MR-Egger intercept was used to indicate directional pleiotropy. In addition, funnel plots were performed for the identification and visualization of extreme outliers.

To examine the potential influence of pleiotropy among the IVs, we searched the genetic variants used as IVs for their potential secondary phenotypes from GWAS Catalog (https://www.ebi.ac.uk/gwas/, last accessed on April 23, 2022). Genetic variants associated with other traits at genome-wide significance were excluded and MR analyses were performed again.

Since both the exposure and outcome datasets that we used included samples from UK Biobank within their meta-analyses, we estimated the bias and type 1 error rates caused by sample overlap^21^ (Supplementary Table 3). In addition, for SmkInit, AgeSmk, CigDay and SmkCes, IVs were selected from the same GWAS meta-analyses excluding participants from UK Biobank (*P* < 5 × 10^–8^, r^2^ < 0.1). Therefore, a total of 22, 1, 27 and 9 genetic variants associated with SmkInit, AgeSmk, CigDay and SmkCes were used as IVs and MR analyses were performed as one of sensitivity analyses, respectively (Supplementary Table 4).

A two-tailed* P*-value < 0.05 was considered statistically significant, unless otherwise noted.

### Ethical approval and informed consent

Our study was approved by the Ethical Committee of Zhejiang Chinese Medical University on Nov 16, 2021 (No. AF-20211116-1). Because the project was mainly based on statistical analyses of publicly accessible databases and published papers, in which informed consent and ethical review were completed separately in each study, we have got the ethical waiver for the whole project.

## Results

The results of F-statistics for IVs used for smoking related phenotypes are presented in Supplementary Table 5. All the F-statistics are much greater than 10, which indicated that the IVs used are less likely to suffer from weak instrument bias.

**Figure 2:**
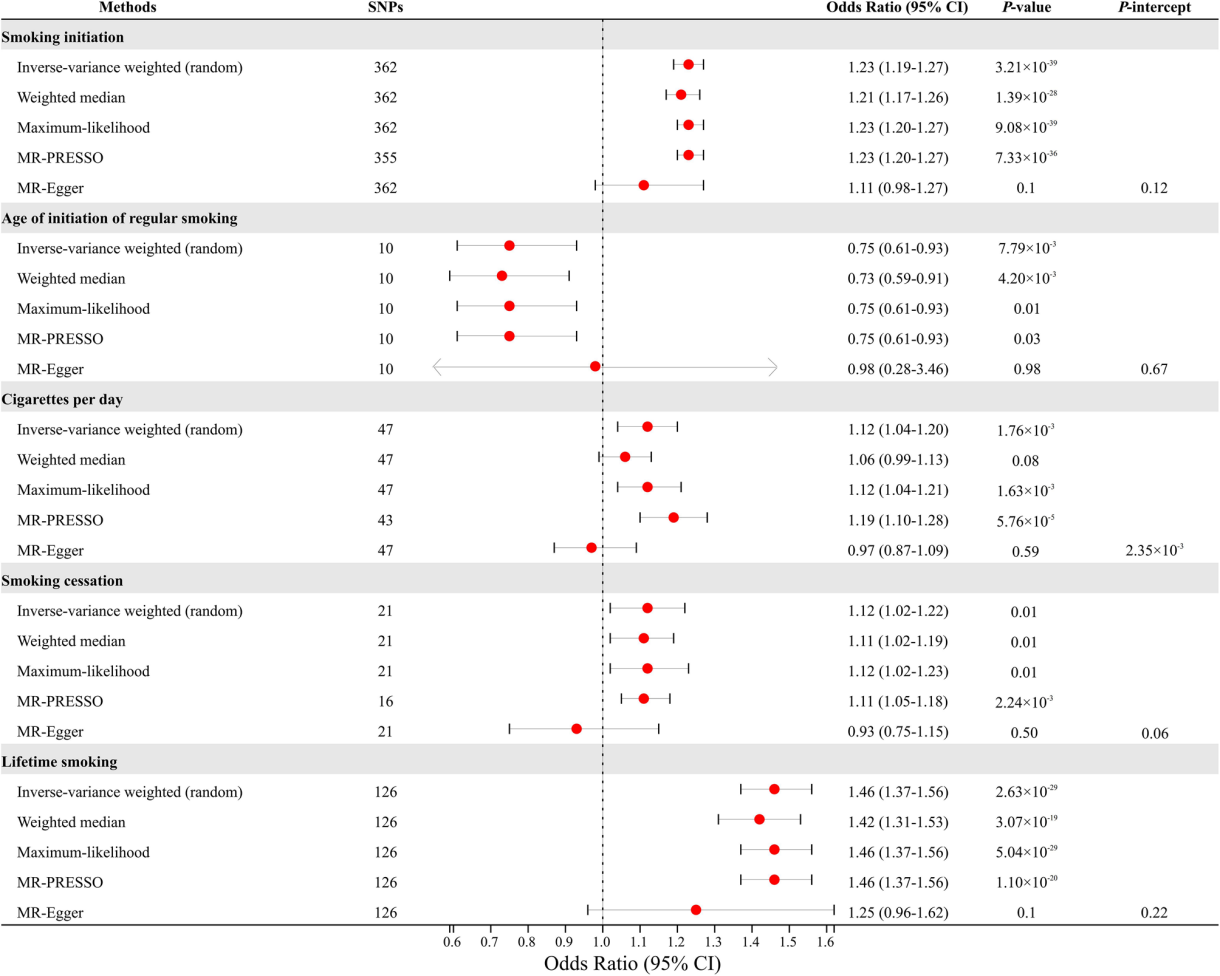
Forest plot of MR analysis of five smoking phenotypes with risk of frailty in ageing.

**Figure 3:**
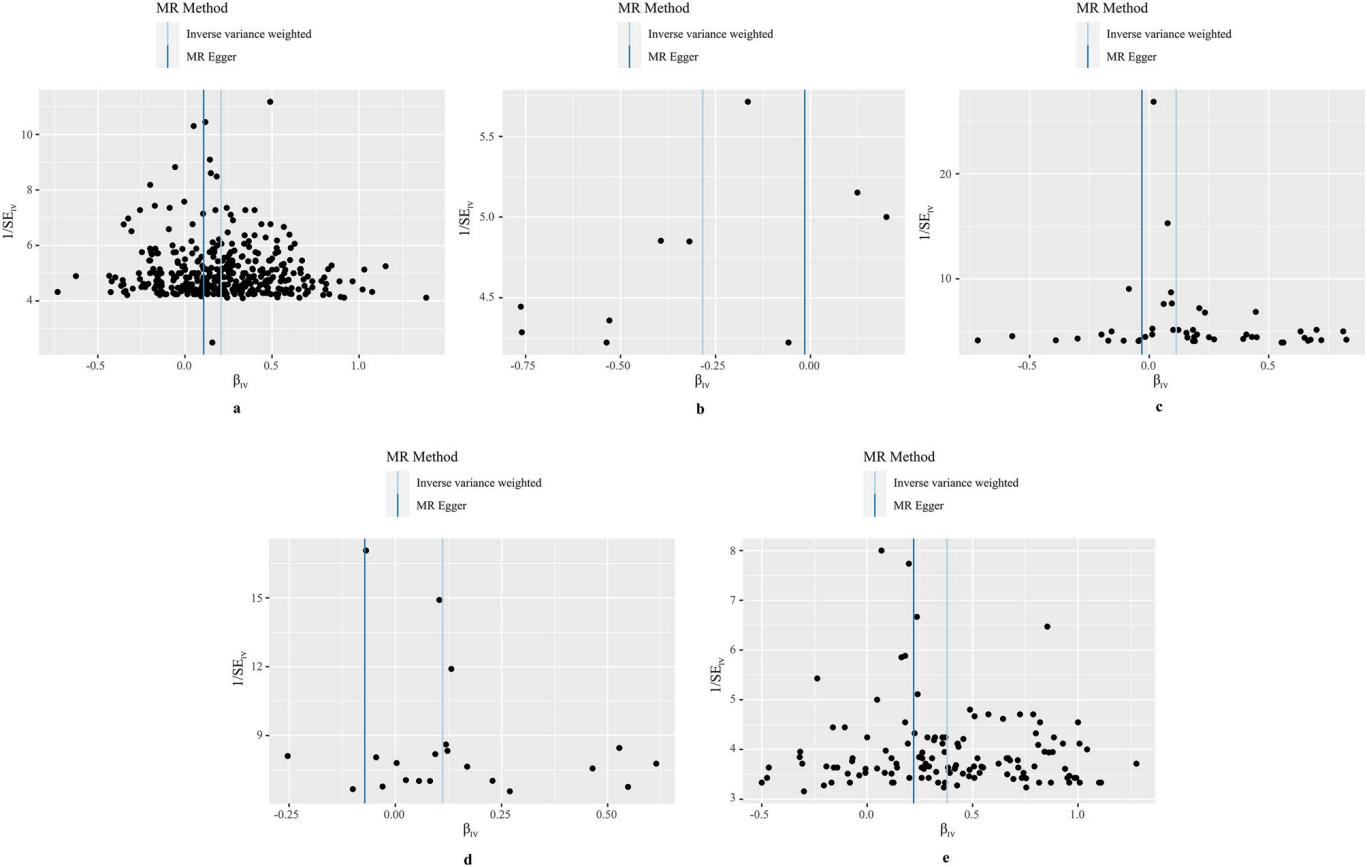
Funnel plots of MR analysis of five smoking phenotypes and frailty in ageing. (**a**) Smoking initiation (SmkInit); (**b**) age of initiation of regular smoking (AgeSmk); (**c**) cigarettes per day (CigDay); (**d**) smoking cessation (SmkCes); (**e**) lifetime smoking.

Figure 2 shows the results of the primary MR analysis for the five smoking related phenotypes with the risk of frailty in ageing. And Fig. 3 shows the funnel plots of five smoking related phenotypes and frailty in ageing. The detailed information of heterogeneity statistics from IVW methods and directional pleiotropy from MR-Egger regression are listed in Supplementary Tables 6 and 7, respectively.

Compared with non-smokers, genetically predicted smoking initiation was positively associated with an increased risk of frailty in ageing (OR 1.23, 95% CI 1.19–1.27, *P* = 3.21 × 10^–39^ by IVW). Results were similar in the weighted median (OR 1.21, 95% CI 1.17–1.26, *P* = 1.39 × 10^–28^) and maximum-likelihood methods (OR 1.23, 95% CI 1.20–1.27, *P* = 9.08 × 10^–39^). Although funnel plot did not identify extreme outliers, seven outliers were found using MR-PRESSO test. Exclusion of the outliers did not change the results essentially (OR 1.23, 95% CI 1.20–1.27, *P* = 7.33 × 10^–36^). MR-Egger regression did not suggest the possibility of pleiotropy (*P* intercept = 0.12).

Consistently, per year increase in genetically predicted age of initiation of regular smoking was associated with a 25% decrease in the risk of frailty (OR 0.75, 95% CI 0.61–0.93, *P* = 7.79 × 10^–3^ by IVW). The effect estimates from the weighted median (OR 0.73, 95% CI 0.59–0.91, *P* = 4.20 × 10^–3^) and maximum-likelihood methods (OR 0.75, 95% CI 0.61–0.93, *P* = 0.01) were similar. No pleiotropic outliers were found in MR-PRESSO test and funnel plot, and MR-Egger regression did not show evidence of directional pleiotropy (*P* intercept = 0.37).

In addition, genetically predicted cigarettes per day (CigDay) was associated with an increased risk of frailty in ageing (OR 1.12, 95% CI 1.04–1.20, *P* = 1.76 × 10^–3^ by IVW). Similar association was observed using the maximum-likelihood methods (OR 1.12, 95% CI 1.04–1.21, *P* = 1.63 × 10^–3^). However, the association became statistically insignificant using the weighted median method (OR 1.06, 95% CI 0.99–1.13, *P* = 0.08). Funnel plot did not suggest the evidence of extreme outliers. MR-PRESSO test found four outliers, and after removing these outliers, the association remained statistically significant (OR 1.19, 95% CI 1.10–1.28, *P* = 5.76 × 10^–5^). MR-Egger regression suggested possible pleiotropy (*P* intercept = 2.35 × 10^–3^). By manually searching the GWAS Catalog, we found 13 SNPs with documented pleiotropy (Supplementary Table 8). Sensitivity analysis removing these potential pleiotropic IVs yielded consistent association, but the effect estimate was attenuated (OR 1.20, 95% CI 1.07–1.35, *P* = 2.42 × 10^–3^).

Furthermore, compared with former smokers, genetically predicted current smokers were associated with an increased risk of frailty (OR 1.12, 95% CI 1.02–1.22, *P* = 0.01 by IVW). Results were similar using the weighted median (OR 1.11, 95% CI 1.02–1.19, *P* = 0.01) and maximum-likelihood methods (OR 1.12, 95% CI 1.02–1.23, *P* = 0.01). Five outliers were found in MR-PRESSO test and after removing these outliers, the association estimate did not change substantially (OR 1.11, 95% CI 1.05–1.18, *P* = 2.24 × 10^–3^). MR-Egger regression did not suggest evidence of potential pleiotropy (*P* intercept = 0.06), and funnel plot did not identify extreme outliers.

Finally, we found that per standard deviation (SD) increase in genetically predicted lifetime smoking was associated with a 46% (95% CI 37%-56%) increased risk of frailty in ageing (*P* = 2.63 × 10^–29^ by IVW). Similar results were obtained using the weighted median (OR 1.42, 95% CI 1.31–1.53, *P* = 3.07 × 10^–19^), maximum-likelihood methods (OR 1.46, 95% CI 1.37–1.56, *P* = 5.04 × 10^–29^), and MR-PRESSO test (OR 1.46, 95% CI 1.37–1.56, *P* = 1.10 × 10^–20^). MR-Egger regression did not suggest evidence of directional pleiotropy (*P* intercept = 0.22). No outliers were found by funnel plots.

As sensitivity analyses, we scanned the genetic variants used as IVs for its other associated phenotypes from the GWAS Catalog. For SmkInit, AgeSmk, CigDay, SmkCes and lifetime smoking, we found a total of 259, 8, 34, 15 and 94 genetic variants used as IVs were associated with other traits, respectively. All the F-statistics were greater than 10 (Supplementary Table 9).After removing these potential pleiotropic variants, the associations did not change essentially (OR 1.21, 95% CI 1.16–1.25, *P* = 4.09 × 10^–22^ for SmkInit; OR 0.77, 95% CI 0.61–0.98, *P* = 0.04 for AgeSmk; OR 1.20, 95% CI 1.07–1.35, *P* = 2.42 × 10^–3^ for CigDay; OR 1.12, 95% CI 1.05–1.18, *P* = 3.64 × 10^–4^ for SmkCes; OR 1.47, 95% CI 1.36–1.58, *P* = 7.67 × 10^–23^ for lifetime smoking) (Supplementary Table 10).

In addition, to avoid the bias caused by sample overlap, we selected IVs for SmkInit, AgeSmk, CigDay and SmkCes from the same GWAS meta excluding participants from UK Biobank and performed MR analysis. We found that the associations between smoking phenotypes and frailty remained statistically significant, except for AgeSmk (OR 1.33, 95% CI 1.23–1.44, *P* = 2.10 × 10^–12^ for SmkInit; OR 1.04, 95% CI 1.01–1.08, *P* = 0.02 for CigDay; OR 1.07, 95% CI 1.02–1.12, *P* = 7.84 × 10^–3^ for SmkCes; OR 0.99, 95% CI 0.73–1.34, *P* = 0.94 for AgeSmk) (Supplementary Table 11).

## Discussion

This study systematically analyzed the potential causal associations of five smoking related phenotypes with the risk of frailty in ageing, and indicated that genetically predisposition to smoking is associated with an increased risk of frailty. To the best of knowledge, this study is the first MR study that provided genetic support for the association between smoking and the risk of frailty in ageing.

These results were generally consistent with former observational epidemiological studies. For example, a cohort study of 94,550 Chinese older people aged 65 years old conducted by Yu et al. reported an increased association between smoking and FI (OR 0.0036, 95% CI 0.0027–0.0045)^22^. Similarly, another cohort study of 980 participants aged 65 years conducted in Canada reported smoking was associated with poorer health status captured by FI and higher mortality rates in both men and women^23^. And an analysis from the Mexican Health and Aging Study including 14,025 individuals ≥ 50 years found that smoking cessation could decrease mortality event for elderly with higher FI (hazard ratio (HR):0.68, 95% CI 0.57–0.80)^24^.

Although our analyses suggested a potential causal role of smoking in the risk of frailty along with the observational evidence, the biological mechanisms underlying the relationship between smoking and in ageing have not been fully understood. There are several possible explanations. As frailty of ageing was measure by FI based on the health deficits accumulation, and smoking can lead to the emergence and development of many tissue injuries and diseases. For example, cigarette smoke as an inflammogen can cause the influx and activation of inflammatory cells^25^. Inflammation which can be measured as high levels of inflammatory markers such as Interleukin-6 (IL-6) and C-reactive protein (CRP) was associated with poor physical performance and muscle strength in older people^26^. In addition, a study identified that smoking depressed muscle protein synthesis and induced an elevated expression of myostatin and muscle atrophy F-box (MAFBx) which were associated with inhibition of muscle growth and muscle catabolism^27^. Besides, smoking is also a major risk factor for cardiovascular^28^ and respiratory diseases^29^, which may greatly affect the health and mortality of the older people.

The present study and all evidence above indicated that smoking is pernicious to the health of frailty in ageing, and smoking cessation could substantially reduce the risk of frailty and decrease the mortality of the elderly. However, most researches and government policies focused on adolescents or the overall adult population^30–32^ rather than older adults. Little attention has been paid to the benefits of smoking cessation for the elderly, doctors are more willing to provide advice of smoking cessation to young patients rather than to older patients^33^, and older smokers also lack motivation for smoking cessation^34^. Therefore, it is of great significance to generate evidence on the importance of smoking cessation for frailty in ageing and provide a reference for government policymaker and medical advice.

This study had several strengths. First, we systematically analyzed five smoking related phenotypes with the risk of frailty and obtained consistent associations using different MR methods and IV sets across five smoking phenotypes. Second, we used a two-sample MR design which overcomes the limitations of confounding bias and reverse causation inherent in conventional observational studies. Third, the genetic variants used in this study were from a meta-analysis of over 30 GWASs including 1,232,091 participants and a GWAS including 462,690 participants, and the outcome datasets of frailty was obtained from a GWAS meta-analysis including 175,226 participants, which yielded adequate statistical validity to estimate causality. There are also some limitations to this study. First, our analyses used the outcome data and IVs European populations, therefore, whether the results of this study can be applied to populations with other ethnics remains uncertain. Second, we could not conduct stratified analysis or investigate nonlinear associations due to the utilization of summary-data level in two sample MR analysis. Third, the datasets used in this study have sample overlap (via UK Biobank) in both exposure and outcome, which may lead to increased type 1 error and bias. However, we found that the type 1 error rates were not increased and the bias was very small, and the results of sensitivity analysis excluding participants from UK Biobank kept stable except AgeSmk, which had only one SNP used as IV. Fourth, the causal relationship between smoking and frailty in ageing may be mediated through other pathways, however, the results were robust in sensitivity analyses.

## Conclusions

In summary, our study indicated that genetically predisposition to smoking was associated with risk of frailty in ageing, which supports a potential causal role of smoking in the risk of frailty. Further studies are needed to elucidate the underlying biological mechanism.

## Supplementary Information

Supplementary Tables.

## Data Availability

Publicly available datasets were used in this study. These can be found at https://www.ebi.ac.uk/gwas/.
